# Supplementary material for: Relationship between adiponectin multimer levels and subtypes of cerebral infarction
Source: PLoS One. 2022 Jan 27;17(1):e0262542. doi: 10.1371/journal.pone.0262542 (PMC8794129; doi:10.1371/journal.pone.0262542)
Supplement: S1 Table — (PDF) [file pone.0262542.s001.pdf]

				(yr)	days	adiponectin (μg/mL)				(score)	(kg/m^2)	(mmHg)	(mmHg)	cholesterol (mg/dL)			(mg/dL)	(mg/dL)
No.	disease	group	sex	Age	from onset	Total	HMW	MMW	LMW	mRS	BMI	sBP	dBP	Total	LDL	HDL	TG	hs-CRP
1	CI	AI	M	73	9	6.66	3.46	1.27	1.94	3		173	79	190	122	49	95	0.10
2	CI	AI	M	77	0	2.27	0.60	0.68	0.99	4	30.5	150	83	176	81	60	173	0.46
3	CI	AI	M	76	0	7.48	3.98	1.42	2.08	3	22.8	172	88	242	158	37	234	2.25
4	CI	AI	M	71	0	4.09	1.34	0.75	2.00	.	22.2	134	83	180	113	42	126	0.57
5	CI	AI	M	70	0	4.47	1.31	1.35	1.81	2	27.4	173	97	200	118	61	106	0.10
6	CI	AI	M	51	1	3.58	1.17	1.25	1.15	3	31.1	208	128	276	194	47	173	1.99
7	CI	AI	M	87	14	9.04	4.28	2.24	2.53	5	20.9	116	71	221	138	64	97	33.2
8	CI	AI	M	74	2	2.36	0.29	0.58	1.49	2	23.9	164	84	205	119	58	141	1.01
9	CI	AI	M	87	2	3.96	1.19	1.00	1.78	0	21.1	163	89	156	71	74	57	7.32
10	CI	AI	M	92	1	11.86	5.41	2.23	4.21	0	17.4	172	78	161	69	78	68	1.75
11	CI	AI	M	73	4	2.62	0.56	0.44	1.62	1	20.0	133	83	182	126	39	87	0.51
12	CI	AI	M	61	4	5.43	2.61	0.72	2.10	4	23.9	115	62	173	111	46	78	51.6
13	CI	AI	M	90	1	5.23	2.58	0.98	1.67	2		159	71	180	113	52	74	3.39
14	CI	AI	M	68		6.46	3.39	1.52	1.56	3	22.2	153	129	144	84		187	6.07
15	CI	AI	M	86	0	11.68	7.22	1.13	3.32	5	20.2	162	95	193	103	76	71	0.76
16	CI	AI	M	61	9	3.77	1.66	0.60	1.51	0		193	94	188	83	61	222	1.20
17	CI	AI	M	83	1	5.00	2.51	1.40	1.09	1	31.6	122	59	173	113	42	89	0.75
18	CI	AI	M	66	1	4.41	1.93	1.07	1.41	3	23.2	161	102	161	97	33	154	0.05
19	CI	AI	M	60	1	3.37	1.45	0.77	1.16	3	33.3	163	97	251	159	45	236	1.12
20	CI	AI	M	76	1	8.05	5.71	1.99	0.36	0	24.7	156	74	127	76	38	63	0.63
21	CI	AI	M	88	0	7.95	4.01	1.38	2.56	3	26.6	105	62	203	128	51	119	4.36
22	CI	AI	M	60	3	3.76	1.79	0.99	0.98	2	19.6	120	84	223	141	47	177	0.26
23	CI	AI	M	59	1	4.94	2.53	0.58	1.83	0	22.4	168	74	164	83	61	99	1.04
24	CI	AI	M	73	0	2.75	0.98	0.42	1.34	0	29.7	161	86	246	168	50	138	0.57
25	CI	AI	M	61	1	4.38	2.20	0.52	1.66	0	22.0	147	88	157	96	40	107	1.10
26	CI	AI	M	74	1	1.42	0.62	0.29	0.51	1	21.9	150	79	186	100	41	223	0.61
27	CI	AI	M	79	2	4.33	1.47	1.14	1.72	1	22.3	150	80	203	114	75	68	3.17
28	CI	AI	M	72	3	3.10	1.31	0.91	0.89	0	24.2	200	108	180	120	31	146	4.56
29	CI	AI	M	68	1	4.08	1.50	1.22	1.36	0	26.7	148	92	238	143	37	292	4.52
30	CI	AI	M	70	28	10.94	6.42	2.98	1.54	2	28.6	185	96	264	166	39	297	0.62
57	CI	AI	M	38	2	2.79	1.12	0.12	1.54	4	25.1	158	109	315	226	51	191	0.94
31	CI	AI	F	77	0	8.04	3.64	1.94	2.45	5	19.7	174	92	206	138	52	79	0.38
32	CI	AI	F	66	1	12.94	7.76	2.98	2.20	0	25.5	170	83	268	173	71	122	0.47
33	CI	AI	F	83	2	15.33	7.94	4.08	3.30	3	20.3	174	73	219	112	88	93	3.14
34	CI	AI	F	85	0	8.20	3.98	1.61	2.61	3	21.7	162	61	155	83	54	91	0.05
35	CI	AI	F	96	2	7.33	3.45	0.85	3.03	4	16.4	172	52	193	126	44	114	0.56
36	CI	AI	F	85	0	10.86	5.46	2.38	3.02	5	23.3	207	83	356	242	63	253	0.73
37	CI	AI	F	78	1	5.22	2.38	1.22	1.62	.	21.8	178	67	138	58	75	26	46.5
38	CI	AI	F	65	0	6.59	2.86	0.27	3.46	2	22.4	124	84	203	113	75	73	0.07
39	CI	AI	F	79	1	13.69	7.88	3.41	2.39	4	22.7	168	73	210	118	75	84	69.5
40	CI	AI	F	76	0	5.35	2.16	1.03	2.16	4	24.5	124	80					1.62
41	CI	AI	F	.	0	9.33	5.38	0.97	2.98	1	21.3	129	78	299	183	58	288	0.47
42	CI	AI	F	84	7	11.57	5.54	1.92	4.11	4	18.6	140	76					0.92
43	CI	AI	F	87	1	8.40	4.69	1.40	2.31	5		176	101	198	112	77	43	12.4
44	CI	AI	F	73	1	11.03	5.28	2.41	3.34	2	23.6	144	88	216	125	73	37	1.00
45	CI	AI	F	73	4	14.34	8.66	2.51	3.17	0	20.4	155	83	249	138	98	64	0.59
46	CI	AI	F	77	0	16.49	9.43	5.18	1.88	4	18.7	149	85	205	134	54	85	7.43
47	CI	AI	F	84	1	6.42	3.41	0.96	2.04	4	19.4	106	71	296	178	107	55	2.63
48	CI	AI	F	61	9	10.66	4.19	4.30	2.16	4	27.3	188	98	215	104	73	281	43.3
83	CI	AI	F	74	0	9.01	4.92	0.73	3.36	4	25.0	158	108	285	208	52	123	1.11
96	CI	CE	M	58	0	11.14	6.06	0.76	4.32	6	22.5	145	109					3.05
97	CI	CE	M	54	19	6.63	3.01	1.52	2.09	1	25.7	129	92	191	116	54	103	0.22
98	CI	CE	M	68	0	7.12	3.23	0.06	3.84	0	20.6	128	86	277	103	156	89	0.89
99	CI	CE	M	78	1	6.19	2.19	1.31	2.69	2	22.3	157	88	218	145	53	101	0.77
100	CI	CE	M	90	0	9.90	3.94	1.73	4.24	5	17.6	143	93	164	89	49	128	0.09
101	CI	CE	M	78	1	8.52	4.04	1.12	3.36	2	20.6	188	113	159	82	66	55	0.54
102	CI	CE	M	91	0	8.11	3.54	1.66	2.92	4	17.9	170	100	191	112	69	48	0.10
103	CI	CE	M	80	1	2.72	0.93	0.54	1.24	0	26.0	133	72	174	100	50	118	0.13
104	CI	CE	M	68	6	3.23	1.11	0.24	1.87	4	23.5	181	126	291	213	56	108	4.85
105	CI	CE	M	74	1	4.61	2.19	0.46	1.96	5	15.5	230	120	284	208	64	59	1.69
106	CI	CE	M	79	2	3.28	0.93	0.48	1.87	1	21.1	171	84	292	218	54	101	2.62
107	CI	CE	M	64	2	5.83	2.87	0.93	2.03	3	22.6	140	115	223	133	77	63	0.88
108	CI	CE	M	74	0	22.47	12.46	4.64	5.36	3	22.0	136	101	131	75		41	8.76

				(yr)	days	adiponectin (μg/mL)				(score)	(kg/m^2)	(mmHg)	(mmHg)	cholesterol (mg/dL)			(mg/dL)	(mg/dL)
No.	disease	group	sex	Age	from onset	Total	HMW	MMW	LMW	mRS	BMI	sBP	dBP	Total	LDL	HDL	TG	hs-CRP
109	CI	CE	M	69	0	8.42	4.91	0.43	3.08	2	20.9	190	95	223	145	63	76	1.68
110	CI	CE	M	77	1	8.56	4.25	0.72	3.59	1	23.9	165	87	220	143	69	41	0.27
111	CI	CE	M	76	0	6.51	2.28	0.21	4.02	5	23.8	141	81	164	93	45	132	1.93
112	CI	CE	M	68	0	5.89	3.31	1.04	1.55	0	23.9	127	62	222	128	86	42	0.33
113	CI	CE	M	75	0	7.24	3.08	0.86	3.31	1	26.7	165	79	170	93	52	127	0.29
114	CI	CE	M	79	13	3.87	1.69	0.48	1.70	6	28.4	170	89					7.84
115	CI	CE	M	70	26	12.41	5.83	1.89	4.70	3	21.4	160	81	190	106	65	94	1.74
116	CI	CE	F	77	0	14.46	7.44	2.87	4.14	6	21.9	144	76	199	123	64	60	0.18
117	CI	CE	F	88	1	11.77	6.62	1.73	3.43	4	20.4	175	83	170	92	70	41	0.39
118	CI	CE	F	73	0	18.33	9.44	4.71	4.19	2	20.0	167	97	208	123	69	79	3.42
119	CI	CE	F	92	0	8.64	4.90	1.90	1.84	4	21.4	143	86	186	107	65	68	5.31
120	CI	CE	F	67	1	7.69	3.24	0.39	4.07	4	23.5	184	89	182	120	48	71	8.46
121	CI	CE	F	78	8	7.39	3.29	0.66	3.44	1	29.9	152	69		110			1.21
122	CI	CE	F	66	2	4.76	2.32	1.12	1.33	4	18.7	136	82	163	99	51	64	15.7
123	CI	CE	F	82	14	15.11	9.39	3.12	2.60	5	18.5	199	97	171	90	67	72	1.74
124	CI	CE	F	79	1	10.11	5.44	0.79	3.88	2	25.3	126	64	188	83	75	150	0.46
125	CI	CE	F	81	1	12.89	7.37	2.92	2.60	4	21.5	149	88	315	214	66	175	0.60
126	CI	CE	F	89	1	8.44	3.99	2.20	2.24	4	21.4	152	84	157	65	66	128	
127	CI	CE	F	91	0	15.91	10.26	2.26	3.38	4	17.3	178	82	188	131	45	62	4.21
128	CI	CE	F	47	1	9.56	6.10	1.13	2.33	2		130	60					0.04
129	CI	CE	F	90	3	22.28	14.23	4.09	3.96	3	18.2	178	82	224	124	85	77	1.96
130	CI	CE	F		4	3.92	2.08	0.58	1.26	0	21.8	102	79	200	116	58	129	1.29
131	CI	CE	F	87	0	20.28	13.27	5.42	1.59	2	22.2	143	80	170	89	62	95	0.49
132	CI	CE	F	76	0	14.81	10.19	0.17	4.45	5	22.0	103	70	208	105	81	112	2.87
49	CI	LI	M	63	5	3.97	2.07	0.85	1.05	1	24.5	182	100	249	163	55	154	0.69
50	CI	LI	M	76	3	6.15	2.94	1.36	1.85	6	18.3	164	78	137	71	39	133	38.00
51	CI	LI	M	68	7	17.47	9.48	1.72	6.28	5	18.4	137	59	145	90	43	62	0.0
52	CI	LI	M	69	2	2.93	0.74	0.22	1.97	.	24.7	176	110	192	112	23	287	0.43
53	CI	LI	M	59	1	5.21	2.19	0.60	2.42	1	22.5	168	105	234	154	61	94	0.27
54	CI	LI	M	62	0	3.30	1.40	0.32	1.58	4	27.0	188	92	182	124	40	88	10.4
55	CI	LI	M	61	2	4.98	2.05	0.55	2.38	1	26.8	154	80	225	145	37	213	5.12
56	CI	LI	M	56	0	3.59	1.30	0.29	2.00	2	27.7	164	107	237	115	51	159	8.48
58	CI	LI	M	75	0	6.82	2.79	0.70	3.33	1	23.6	150	79	242	146	43	265	0.44
59	CI	LI	M	72	3	6.91	3.24	0.72	2.95	2	23.7	167	79	208	116	45	233	0.12
60	CI	LI	M	73	6	8.54	4.70	0.42	3.42	1	24.3	176	96	205	136	48	107	1.51
61	CI	LI	M	80	21	9.26	3.55	1.69	4.02	1	21.0	149	62	129	36	67	130	0.59
62	CI	LI	M	74	0	5.89	2.76	0.16	2.98	1	22.6	152	77	219	123	71	127	0.41
63	CI	LI	M	57	1	5.21	2.77	0.62	1.82	1	24.3	151	84	143	83	.	136	3.17
64	CI	LI	M	65	4	4.98	2.48	0.36	2.14	1	25.1	146	90	207	73	59	374	0.36
65	CI	LI	M	85	1	6.72	3.55	0.48	2.70	2	22.1	140	73	227	150	57	102	7.97
66	CI	LI	M	78	0	7.66	3.89	0.28	3.49		22.1	193	110	245	143	37	327	0.57
67	CI	LI	M	70	1	3.86	1.71	0.11	2.03	0	24.5	180	107	235	158	56	105	0.37
68	CI	LI	M	85	8	5.89	3.58	0.08	2.24	4	21.0	154	74	123	61	41	107	2.27
69	CI	LI	M	74	17	11.62	5.55	2.02	4.04	4	26.9	165	73	201	111	78	61	46.0
70	CI	LI	M	64	1	5.29	2.60	0.39	2.30	1	23.0	156	82	311	207	64	199	0.53
71	CI	LI	M	76	12	7.43	4.76	0.52	2.14	5	16.2	154	91	212	116	86	50	4.05
72	CI	LI	M	82	0	23.32	16.11	0.92	6.29	4	20.3	162	64	192	101	77	72	0.96
73	CI	LI	M	77	2	7.25	3.08	0.77	3.40	2	18.1	138	66	251	142	95	70	55.4
74	CI	LI	M	69	2	4.81	2.22	0.65	1.94	5	26.3	173	87	181	137	26	92	0.94
75	CI	LI	M	62	1	4.61	1.94	0.03	2.65	1	22.7	140	78	191	110	58	117	0.20
76	CI	LI	M	81	2	6.17	2.96	0.06	3.15	4	23.4	183	101	202	138	43	107	8.44
77	CI	LI	M	83	0	8.20	4.52	0.53	3.14	4	25.0	173	75	195	133	40	109	0.98
78	CI	LI	F	82	3	6.82	3.22	1.36	2.23	4	23.3	138	73	177	105	49	114	4.12
79	CI	LI	F	89	1	12.67	8.16	0.59	3.92	1	24.4	136	92	153	77	58	89	0.25
80	CI	LI	F	71	0	20.97	12.53	3.31	5.13	2	18.0	153	99	191	109	64	88	0.23
81	CI	LI	F	87	4	5.28	3.59	0.67	1.03	4	21.8	186	93	193	123	49	103	0.44
82	CI	LI	F	77	0	7.47	3.93	1.08	2.46	4	28.4	130	80	233	152	65	79	0.29
84	CI	LI	F	74	0	5.77	2.72	0.37	2.69	1	25.7	180	72	225	118	82	126	.
85	CI	LI	F	60	0	6.73	2.66	0.57	3.50	0	25.9	178	105	268	185	60	116	0.28
86	CI	LI	F	87	0	16.58	9.12	4.37	3.09	5	17.4	139	55					0.45
87	CI	LI	F	78	2	10.53	6.59	0.78	3.17	3	26.1	144	81	239	153	61	127	0.21
88	CI	LI	F	49	3	14.74	7.88	1.38	5.48	1	19.4	151	92	346	238	40	340	0.07
89	CI	LI	F	76	1	17.09	10.30	3.55	3.23	4	25.6	176	89	211	128	71	61	0.13

				(yr)	days	adiponectin (μg/mL)				(score)	(kg/m^2)	(mmHg)	(mmHg)	cholesterol (mg/dL)			(mg/dL)	(mg/dL)
No.	disease	group	sex	Age	from onset	Total	HMW	MMW	LMW	mRS	BMI	sBP	dBP	Total	LDL	HDL	TG	hs-CRP
90	CI	LI	F	88	1	21.14	13.75	2.45	4.94	2	19.0	133	75	224	120	96	41	0.65
91	CI	LI	F	80	1	6.09	2.95	1.02	2.13	4		174	86	218	130	71	85	1.44
92	CI	LI	F	68	1	4.09	1.79	0.55	1.74	4	29.3	167	105	171	115	45	57	0.93
93	CI	LI	F	84	1	8.01	3.95	0.94	3.12	5		154	79	214	146	44	118	1.28
94	CI	LI	F	97	2	10.84	7.19	0.48	3.17	5		186	91	149	80	33	181	1.46
95	CI	LI	F	84	1	11.62	4.83	2.69	4.10	1	25.9	210	95	214	109	79	132	0.66

CI, cerebral infraction; M, male; F, female; HMW, high molecular weight; MMW, medium molecular weight; LMW, low molecular weight; mRS, modified Rankin scale; BMI, body mass index; sBP, systolic blood pressure; dBP, diastolic blood pressure; TG, triglycerides; hs-CRP, high sensitive
