# Supplementary material for: Relationship between adiponectin multimer levels and subtypes of cerebral infarction
Source: PLoS One. 2022 Jan 27;17(1):e0262542. doi: 10.1371/journal.pone.0262542 (PMC8794129; doi:10.1371/journal.pone.0262542)
Supplement: S2 Table — (PDF) [file pone.0262542.s002.pdf]

Atherothrombotic infarction (AI)

	male				female				
adiponectin (μg/mL)	n	Mean	±	SD	n	Mean	±	SD	
Total	31	5.23	±	2.76	19	10.04	±	3.36	***
HMW	31	2.47	±	1.81	19	5.21	±	2.18	***
MMW	31	1.09	±	0.63	19	2.11	±	1.36	***
LMW	31	1.67	±	0.76	19	2.71	±	0.65	

Lacunar infarction (LI)

	male				female				
adiponectin (μg/mL)	n	Mean	±	SD	n	Mean	±	SD	
Total	28	7.07	±	4.30	17	10.97	±	5.46	*
HMW	28	3.61	±	2.96	17	6.19	±	3.65	*
MMW	28	0.62	±	0.52	17	1.54	±	1.25	*
LMW	28	2.85	±	1.22	17	3.24	±	1.20	

Cerebral embolism (CE)

	male				female				
adiponectin (μg/mL)	n	Mean	±	SD	n	Mean	±	SD	
Total	20	7.63	±	4.35	17	12.14	±	5.27	**
HMW	20	3.59	±	2.55	17	7.03	±	3.67	**
MMW	20	1.05	±	1.00	17	2.12	±	1.56	**
LMW	20	2.99	±	1.18	17	2.98	±	1.08	

HMW, high molecular weight; MMW, medium molecular weight; LMW, low molecular weight; n, number of data; differrence in levels between sex was indicated by *, $p < 0.05$; **, $p < 0.01$; ***, $p<0.001$.
