# Supplementary material for: Relationship between adiponectin multimer levels and subtypes of cerebral infarction
Source: PLoS One. 2022 Jan 27;17(1):e0262542. doi: 10.1371/journal.pone.0262542 (PMC8794129; doi:10.1371/journal.pone.0262542)
Supplement: Attachment — Submitted filename: Response to reviewer 1.docx [file pone.0262542.s003.docx]

Response to Reviewer: 1

Thank you very much for your careful and kind review of our manuscript entitled “Relationship between adiponectin multimer levels and subtypes of cerebral infarction” (Ms. No. PONE-D-21-26718). We have addressed all the comments by reviewers, as indicated on the separate pages, and we hope that the explanations and revisions of our work are satisfactory.

*It is an interesting study that aimed to describe the relationship between adiponectin isoforms and stroke subtypes. There is relevance in the theme because stroke molecular biomarkers is still a controversial field in the literature.*

*Introduction was well written, the problem is clear, however the authors could provide some hypotheses.*

*About methods, I’m a little worried about when volunteers were included in the study. Authors described " a total of 132 patients with cerebral infarction who were enrolled between November 2008 and April 2009 were included in the study".*

1) Blood samples were stored since 2009?

We collected and stored serum samples from subjects since November 2009.

Therefore, we added the following sentence in Methods section.

Line 89

All serum samples from those 132 subjects were stored since November 2008.

2) How could you ensure the sample integrity?

We appreciate your suggestion for sample integrity, and then added the following description.

Line 96-99

Venipuncture blood was directly collected into a tube containing aprotinin, polypeptide protease inhibitor and was immediately centrifuged at 4 ℃. Serum sample was divided into tubes in a 0.1-mL aliquot and stored at -80 ℃ until used.

3) Please, clarify when molecular assays were performed.

We added the following sentence.

Line 89-90

Adiponectin assays were performed during May and Jun in 2013.

4) In addition, please describe if there was a blind evaluator for groups.

We thank for your suggestion to conduct blind study. However, this study was not performed by a blind evaluator.

5) Authors must describe when the samples were collected: 1 hour, 8 hours, 24 hours after stroke… The results can be affected according to time after stroke.

There was no significant difference in the days that serum samples were collected after onset among AI, LI and CE. In each group approximately 80％ of samples were collected within two days after onset (AI:83.7%, LI:75.6% and CE:81.1%) and 4-8% of the samples were collected in more than two weeks after onset (AI:4.1%, LI:6.7% and CE:8.1%).

We added “Day from onset (day)” in each group in Table 1.

Line 91

6) About ELISA, were samples assessed in duplicate or triplicate?

We added “in duplicate” in the text.

Line 100

The serum levels of total and multimeric adiponectin were measured in duplicate using an enzyme-linked immunosorbent assay (ELISA) kit

7) Also describe any method used for stroke image diagnosis.

We added the methods for stroke image diagnosis in the text.

Line 85-86

Clinical characterization of the cerebral infarction was performed using magnetic resonance imaging including T2-weighted imaging (T2WI) and diffusion tensor imaging (DTI).

8) About results, I believe authors should provide the real values of p in the text (Line 120 -Relationship between adiponectin forms and stroke subtypes; Line 153 Correlation between the adiponectin forms and clinical parameters). For figures and tables OK.

We added the real *p* values of each relation in the text.

9) Regarding to discussion, try not to repeat the introduction content.

We deleted the following sentences.

Line 235

The onset of AI is caused by large-artery atherosclerosis associated with metabolic syndrome, which includes hypertension, dyslipidemia, and glucose metabolism disorders. Adiponectin has been shown to exert anti-atherogenic and anti-inflammatory effects [18, 19]. Serum levels of total adiponectin are known to decrease in coronary artery disease [20]. Sasaki *et al.* reported that plasma adiponectin levels were lower in subjects with cerebral infarction than in control subjects [17].

Line 237

HMW adiponectin is thought to be the major active form, but its

Line 237

HMW adiponectin is closely associated with the suppression of the onset of coronary artery disease, weight loss [9], and the effects of insulin sensitizers [10].

10) Please, could you bring more information about biomarkers and functional recovery? I think it´s an important aspect for discussion.

We added the following sentences and references.

Line 283-288

This study examined serum levels of multimeric adiponectin at only one point after onset. Other studies have reported improvement of serum adiponectin levels and functional recovery by post-stroke treatment and rehabilitation [27-30]. Biomarkers should be useful for disease detection, prognosis determination, follow-up, and therapeutic effect determination. It is necessary that comparison of serum multimeric adiponectin levels between after onset and after a period when functional recovery is observed.

11) mRS score needs more attention and interpretation.

We added the following sentences.

Line 268-275

To preliminarily examine the relationship between adiponectin levels and disability or prognosis in cerebral infarction we chose mRS that has been most widely applied for evaluating recovery from stroke and is also commonly used as a functional prognosis evaluation item in clinical studies. However, mRS is based on the subjective evaluation of the evaluator and it is also known that there are considerable fluctuations. In the present study, for consistency of validity and reliability of the mRS, scoring with interview was performed by cerebral stroke specialists.

Line 280-282

Therefore, it is difficult to directly determine the prognosis and dysfunction from the adiponectin levels, and it is necessary to consider various factors and interpret the relation.

12) Some correlations are positive and others negative, could it be related to some type of U effect or increase in adiponectin resistance because of loss of receptors?

Thank you for your suggestion on correlation analysis.

We added the following sentences.

Line 324-331

Analyzing the correlation between adiponectin and various factors is an effective tool for searching for biomarkers. If adiponectin impairs the functional molecular process, it may be directly reflected and predict the diagnosis, prognosis, and course of the disease. In addition, it must be considered that the receptor or signal transduction of adiponectin is impaired. It is also necessary to know that when the optimal concentration range of serum adiponectin or the factor to be examined is narrow, the correlation becomes positive or negative due to abnormally low or abnormally high values.

13) The lack of sensitive tools for measuring body composition as a limitation.

We added the following sentence.

Line 338-341

Visceral fat measurements have been performed as direct methods using computer tomography (CT) and magnetic resonance imaging (MRI) as direct methods, but there are still limitations due to the lack of sensitive tools for measuring body composition.

About “data not shown” (submitted version: line 130)

Line 138

We deleted the description “data not shown”.

Instead, we added supplemental table 2.

About “Data Availability Statement”

We make all data underlying the findings in this manuscript fully available. We present those data as supplemental table 1.

About the measurements for serum multimeric adiponectin levels by ELISA kit,

We added the following sentences.

Line 101-104

Briefly, the adiponectin levels of total, HMW and (HMW and MMW) were measured by this ELISA kit. MMW adiponectin levels were calculated by subtracting HMW from (HMW and MMW). LMW levels were calculated by subtracting (HMW and MMW) from total adiponectin.

We corrected References.
